# Sonic Hedgehog Signaling Promotes Survival and Lineage Specific Differentiation of Transplanted Neural Precursor Cells and Improves Motor Recovery After Thoracic Spinal Cord Injury in Rats

**DOI:** 10.3390/biology15151291

**Published:** 2026-08-04

**Authors:** Mohamed Tail, Guoli Zheng, Hao Zhang, Hao Wang, Anna-Kathrin Harms, Maryam Hatami, Thomas Skutella, Andreas Unterberg, Klaus Zweckberger, Alexander Younsi

**Affiliations:** 1 Department of Neurosurgery, University Hospital Heidelberg, 69120 Heidelberg, Germany; mohamed.tail@med.uni-heidelberg.de (M.T.);; 2 Department of Neurology, University Hospital Heidelberg, 69120 Heidelberg, Germany; 3 Medical Faculty Heidelberg, University of Heidelberg, 69120 Heidelberg, Germany; 4 Department of Neuroanatomy, Institute for Anatomy and Cell Biology, University of Heidelberg, 69120 Heidelberg, Germany; 5 Department of Neurosurgery, BG Klinik Ludwigshafen, 67071 Ludwigshafen, Germany

**Keywords:** spinal cord injury, sonic hedgehog, neural precursor cells, neuroregeneration, neuroinflammation

## Abstract

Transplanted neural precursor cells (NPCs) can support repair after spinal cord injury (SCI) but often face poor survival and limited differentiation within the hostile post-injury microenvironment. We tested whether subacute activation of Sonic hedgehog (Shh) signaling improves NPC engraftment and functional recovery over the duration of 6 weeks. Female Wistar rats underwent T9/T10 clip-contusion SCI and were assigned to vehicle, Shh-only, NPC-only, Shh + NPC, or sham groups (*n* = 8 per SCI arm, *n* = 5 sham, total *n* = 37). GFP + NPCs (4 × 10^5^) were grafted at 7 days post injury. Recombinant Shh or vehicle was delivered intrathecally via osmotic pumps for 7 days thereafter. Outcome measures included pathway activation (GLI1, SMO), NPC differentiation (NeuN, Olig2, Nestin, GFAP), astrogliosis (GFAP), CSPG deposition, immune cell infiltration (CD3, Iba1), apoptosis (cleaved Caspase-3), and locomotion (BBB, Gridwalk, CatWalk) over 6 weeks. Shh delivery increased GLI1/SMO expression and, when combined with NPCs, significantly elevated NPC-derived neurons and oligodendrocytes while preserving undifferentiated NPCs. Combined therapy reduced astrogliosis, CSPG deposition, T-cell and macrophage/microglial densities, cyst size and apoptotic cells compared with controls. These histological benefits were accompanied by improved locomotor scores in BBB, Gridwalk and selected CatWalk parameters. Our findings highlight the synergistic effects of Shh pathway activation and NPC transplantation in promoting neuroregeneration after SCI. The data support Shh as an adjunct to NPC transplantation for SCI repair.

## Simple Summary

Spinal cord injuries often cause permanent loss of movement because the injured spinal cord cannot repair itself and instead forms scar tissue that blocks healing. A promising strategy is to transplant immature stem cells of the nervous system, called neural precursor cells, into the damaged area so that they can replace lost nerve cells. However, most transplanted cells die or fail to mature in the hostile environment of the injured cord. In this study, we tested whether a natural signaling molecule called “Sonic Hedgehog”, which normally guides the development of the nervous system, can help the transplanted cells survive and work better. Using a rat model of spinal cord injury, we combined the transplanted cells with this molecule and followed the animals for six weeks. The combination helped more cells survive and mature into nerve and insulating cells, reduced scarring and inflammation, and improved the animals’ ability to walk.

## 1. Introduction

Spinal cord injury (SCI) causes persistent neurological disability and societal burden, with few therapies that meaningfully restore function [1,2,3]. Transplantation of neural precursor cells (NPCs) remains attractive because NPCs generate neurons and glia, modulate the lesion milieu, and can integrate into host circuits [4,5,6,7]. However, post-transplant survival and lineage maturation are modest in vivo, limiting functional benefit [8,9,10].

The initial mechanical trauma in SCI is followed by a protracted secondary-injury cascade—ischemia, glutamate excitotoxicity, oxidative stress, and sustained neuroinflammation—that propagates tissue loss well beyond the original epicenter [11]. Over the following days to weeks, reactive astrocytes, microglia, and infiltrating immune cells demarcate the lesion and deposit a chondroitin-sulfate-proteoglycan-rich matrix, forming a glial scar that, although it initially contains the damage, matures into a durable barrier to axonal growth and endogenous repair [12,13]. This same cytotoxic, inflammatory, and scar-forming milieu is inhospitable to exogenous cells, contributing to the poor graft survival, limited integration, and skewed differentiation that have tempered the clinical translation of stem- and precursor-cell therapies for SCI [5,10]. These obstacles have shifted emphasis toward combinatorial approaches that pair cell transplantation with agents capable of remodeling the lesion into a more regeneration-permissive environment.

Sonic hedgehog (Shh) regulates ventral cord differentiation and progenitor dynamics via PTCH-SMO-GLI signaling [14,15,16,17]. Beyond development, Shh can modulate astrogliosis and inflammatory tone and has been reviewed as a target for SCI repair across neuroprotection, plasticity, and immune regulation [13,18,19,20]. In rodent SCI, pump-based Shh in the subacute phase reduces neuroinflammation and supports recovery [21,22,23].

In our previous works, we were able to link Shh with increased NPC viability and neuronal differentiation under inflammatory stress in vitro [24], as well as reduction in inflammatory response in vitro after SCI [23]. We chose to design this experiment to test the synergistic effects of local application of Shh and transplantation of NPCs after SCI. In contrast to studies delivering Shh alone or combining Shh with other precursor populations, our study focuses on NPC grafts at a clinically relevant timing and route, aiming to test adjunctive Shh as a microenvironmental priming strategy. Primary endpoints are the effects on survival and fate of transplanted NPCs, as well as functional recovery over 6 weeks. We posited that adjunct Shh + NPC, rather than either alone, would enhance NPC survival and bias fate toward neuronal and oligodendroglial lineages, attenuate astrogliosis and immune cell infiltration, as well as improve locomotion.

## 2. Materials and Methods

### 2.1. Isolation and Culture of NPCs

**Figure 1:**
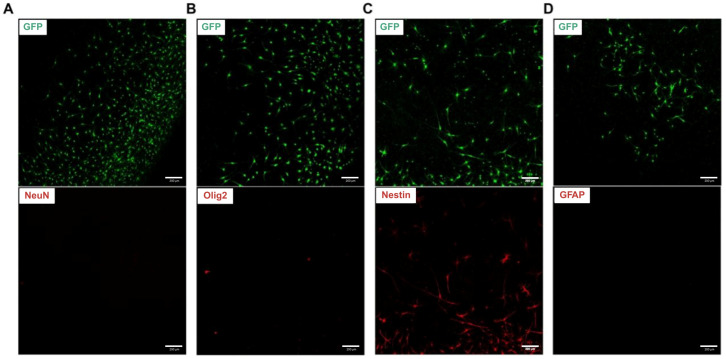
In vitro NPC cultures at 10× magnification. Stained with NeuN (**A**), Olig2 (**B**), Nestin (**C**), and GFAP (**D**). Scale bar: 200 μm.

NPCs were isolated from the subventricular zone (SVZ) of 2-week-old embryos of green fluorescent protein (GFP)-expressing transgenic Wistar rats (Rat Resource & Research Center, University of Missouri, Columbia, MO, USA; strain F344-Tg(UBC-EGFP)F455Rrrrc). The seeding density was controlled at 1.5 × 10^4^ cells/mL. The expansion culture medium consisted of DMEM/F12 medium (Thermo Fisher, Waltham, MA, USA), 1% N2 supplement (Thermo Fisher, Waltham, MA, USA), 1% penicillin/streptomycin (Sigma-Aldrich Inc., St. Louis, MO, USA), 10 ng/mL epidermal growth factor (EGF, E1257, Sigma-Aldrich Inc., St. Louis, MO, USA), and 10 ng/mL basic fibroblast growth factor (bFGF, F0291, Sigma-Aldrich Inc., St. Louis, MO, USA). Before transplantation or assessment, immunohistochemical (IHC) staining was performed to identify stem cell characteristics, viability, and differentiation. As shown in Figure 1, NPCs were negative for the mature markers NeuN (A), Olig2 (B), and GFAP (D), but were predominantly positive for the precursor marker Nestin (C), a marker indicative of an undifferentiated state, prior to transplantation into the injured spinal cord on day 7. The NPC expansion protocol used here was established in our previous work [8,9].

### 2.2. Animals, Experimental Groups, and Study Design

A total of 37 female Wistar rats (160 g; Charles River Laboratories, Sulzfeld, Germany) were split into 5 groups (Table 1). The animals were randomly distributed into 1815 cm^2^ cages (590 × 380 × 200 mm) with 3 or 4 animals per cage and housed under a 12 h light/12 h dark cycle at 26 °C. All animals were randomly assigned to the experimental groups using a computer-generated random number sequence. Testing order was also randomized to minimize confounding factors.

Food and water were provided ad libitum. Enrichment toys were removed from the cages to avoid differences in activity level. All the animals were provided with specific and intensive care for five days, involving analgesic administration, surgical wound control, and manual bladder voiding. Furthermore, the animals were weighed every other day. No autophagia/autotomy, urinary-tract infections, abnormal weight loss, or peri-operative mortality were observed. No animals were excluded from analysis. All the experiments met the requirements of “The Animal Protection Law and Animal Research Regulation of the Federal Republic of Germany”, and permission to perform all the experiments was approved by the Animal Care Committee Karlsruhe, Germany, prior to the study (reference G-68/18).

### 2.3. Surgical Procedures

For all surgical procedures, the animals were anesthetized with isoflurane (3.5% for induction and 1.5% for maintenance). After surgery, analgesics (0.05 mg/kg buprenorphine s.c.; Bayer, Leverkusen, Germany and 2 mg/kg meloxicam s.c.; Boehringer-Ingelheim, Ingelheim, Germany) were administered for five days, and antibiotic prophylaxis (4 mg/kg moxifloxacin p.o.; Alcon, Fort Worth, TX, USA) was given for seven days.

**Figure 2:**
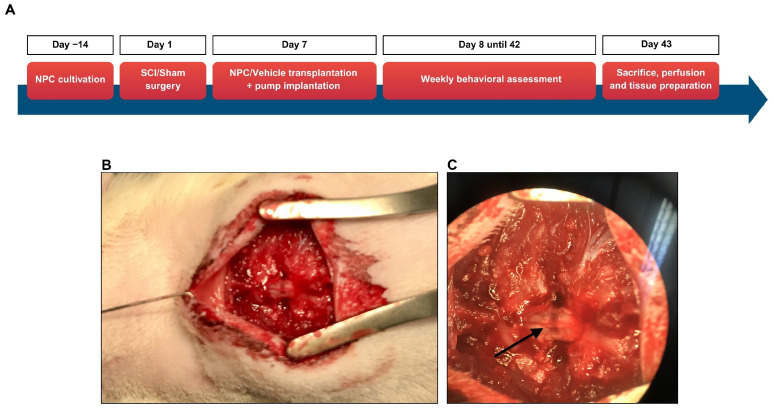
Experimental design and surgical spinal cord injury model. (**A**) Experimental timeline: NPC cultivation (Day −14), SCI/sham surgery (Day 1), NPC/vehicle transplantation and pump implantation (Day 7), weekly behavioral assessment (Days 8–42), and sacrifice, perfusion and tissue preparation (Day 43). (**B**) Day 7, post-SCI, pre-implantation: laminectomy at T9 with the injured spinal cord visible. (**C**) Higher-magnification view of the exposed injured cord (black arrow).

For the animals in all the groups except the sham group, a 28 g modified aneurysm clip (Fehlings Laboratory, Toronto, ON, Canada) was used to generate a contusion and compression SCI at the T9 level; meanwhile, the sham group animals underwent only a T9 laminectomy (Figure 2).

NPC transplantation into the spinal cord via stereotactic injection was performed seven days post-injury (dpi). The previous skin incision was reopened, and the epidural space was carefully re-exposed and dissected through the scar tissue. After the visualization of the previous T9 injury, we used a Hamilton syringe (Hamilton Company, Bonaduz, Switzerland) equipped with a 35 g microneedle (NF35BV-2; World Precision Instruments, Berlin, Germany) to minimize the degree of tissue damage caused by the injection for the stereotactic transplantation of NPCs or the same amount of saline. For the animals in groups 3 (NPC-only) and 4 (Shh + NPC), 8 µL of the NPC suspension containing 4 × 10^5^ cells was injected into the spinal cord at four different positions (2 µL each): 1 mm bilateral to the midline and 2 mm rostral or caudal to the lesion epicenter. The depth of the injection was 1.5 mm, and the speed was controlled at 0.5 µL/min (World Precision Instruments, Berlin, Germany). The needle was placed in the spinal cord for an additional minute to ensure proper diffusion. The other three groups were subjected to the same procedure, except that 8 µL of NPC culture medium without NPCs was added.

Following stereotactic injection, an additional laminectomy of T11 was performed, and an intrathecal microcatheter (Alzet, Cupertino, CA, USA), with one end connected to a subcutaneous osmotic micropump (1007D; Alzet, Cupertino, CA, USA), was inserted subdurally with the other end positioned upon the lesion of T9 SCI. The pumps were filled with either 100 µL of 50 ng/mL Shh (for the Shh and Shh + NPC groups) (R&D Systems, Minneapolis, MN, USA) or 0.9% NaCl (vehicle and NPC-only groups) and were preloaded at room temperature for 2 h [21]. The dose of 50 ng/mL recombinant Shh was selected based on our group’s prior validation in our previous work [21]. Furthermore, in this study, we included analysis of canonical downstream markers GLI1 and SMO to validate pathway activation [22,23]. The pump delivered its contents for seven days at a flow rate of 0.5 µL/h. The sham group animals received the construction of a subcutaneous pocket for the Alzet^®^ pump and a sham laminectomy without dural puncture. As a standard for antibiotic prophylaxis and immunosuppression, minocycline (50 mg/kg s.c.; Ratiopharm, Ulm, Germany) was administered daily from two days prior to 10 days after transplantation, and daily injections of cyclosporin (10 mg/kg s.c.; Sigma-Aldrich, St. Louis, MO, USA) were initiated on the day of transplantation until the end of the experiment in all groups.

### 2.4. Behavioral Testing

The Basso–Beattie–Bresnahan open field score (BBB) was used to evaluate hindlimb locomotion recovery. Hindlimb movement, joint movement, coordination, trunk and tail position, and weight support of the rats were evaluated via a 4 min video [25]. Two overhead cameras at two different angles were installed to ensure that there would be no visual blind spots during the movement of the animals. The videos were reviewed by two blinded observers, and scores were averaged.

The integrity of the descending neuronal tracts controlling fine movements was assessed with the gridwalk test in all the animals. In short, animals need to run through a 1 m long pathway of randomly placed grids. Misplacement of a hindlimb between the bars, defined as the number of stepping errors, was documented and summed over four runs [26]. After the videos were reviewed separately, the number of stepping errors was averaged between two blinded observers.

In the CatWalk XT^®^ automated gait analysis, the animals were placed at one end of the horizontal glass tunnel and voluntarily ran toward the other end. At least three compliant and uninterrupted runs were recorded in one trial for further analysis via CatWalk XT^®^ software (version 10.5; Noldus Information Technology, Wageningen, The Netherlands) [27]. A maximum run variation of 60%, a camera gain of 16.99 decibels (dB), and a detection threshold of 0.1 arbitrary units were set as the default detection configurations for all runs. Each run was automatically classified by the software and then manually reviewed to correct the mislabeling of the paws and remove the redundant contact label from the animal’s nose, abdomen, or tail.

All behavioral tests were carried out one day pre-injury as a baseline control and were repeated weekly until six weeks post-injury.

### 2.5. Immunohistochemical Staining

Animals from each group were sacrificed and perfused with 50 mL of 0.1 M cold phosphate-buffered saline (PBS) followed by 150 mL of 4% paraformaldehyde (PFA) on day 43. The spinal cord was removed at a length of 10 mm centered on the lesion or landmark of the T9 vertebral body. After fixation with PFA for 24 h and cryoprotection in 30% sucrose for 48 h, the spinal cord was embedded in tissue embedding medium (Sakura Finetek Europa B.V., Alphen aan den Rijn, The Netherlands) and cut into 30 µm-thick sections with a cryostat (Leica Biosystems, Nussloch, Germany). All the tissues were dried and stored at −80 °C until further processing.

After application of blocking solution (5% non-fat milk powder, 1% bovine serum albumin, and 0.3% Triton X-100), the spinal cord sections were incubated with the following primary antibodies at 4 °C overnight: anti-NeuN (1:500; ABN78, Millipore, Burlington, MA, USA), anti-Olig2 (1:50; M3821, Sigma-Aldrich, St. Louis, MO, USA), anti-Nestin (1:400; MAB353, Millipore, Burlington, MA, USA), anti-GFAP (1:250; ab53554, Abcam, Cambridge, UK), anti-GLI1 (1:200; sc-515751, Santa Cruz, Dallas, TX, USA), anti-SMO (1:200, sc-166685, Santa Cruz, Dallas, TX, USA), anti-cleaved Caspase-3 (1:400; Asp175, Cell Signaling, Danvers, MA, USA), anti-CD3 (1:200; MCA772, Bio-Rad, Hercules, CA, USA), anti-iba1 (1:200, NB100-1028, Novus, Wiesbaden, Germany), anti-LC3B (1:200, Cell Signaling, Danvers, MA, USA), and anti-CSPG (1:400; MAB1581; Millipore, Burlington, MA, USA). The following day, the sections were incubated with the secondary antibodies (1:500; ab150129, ab175700, ab150075; Abcam, Cambridge, UK) and DAPI (1:10,000; #6335.1; Carl Roth, Karlsruhe, Germany) for one hour before sealing with mounting medium.

### 2.6. Image Analysis

All immunofluorescence images of the stained sections were captured with a confocal laser scanning microscope (LSM 700; Carl-Zeiss, Jena, Germany) with ZEN microscope software (ZEN 2010; Carl Zeiss, Jena, Germany). All observations, image acquisition, and analyses of the sections were conducted under blinded conditions.

For quantitative assessment of chondroitin sulfate glycosaminoglycan (CS-GAG) deposition and astrogliosis, the immunointensity of CSPG and GFAP was measured on eleven distinct cross-sectional images (0 µm,  ±240 µm,  ±480 µm, ±720 µm, ±960 µm and  ±1200 µm from the lesion epicenter) via ImageJ (Version 1.53n, National Institutes of Health; Bethesda, MD, USA). The results were averaged within each animal and displayed in integrated pixel intensity per mm^2^ (pi/mm^2^).

The cross-sectional area of the posttraumatic intramedullary cyst was additionally delineated in each of these eleven GFAP-stained sections using ImageJ, and the resulting areas were averaged per animal and reported as mean cyst area (mm^2^).

To quantify the differentiation fate of NPCs, the cells activated by the Shh pathway, T-lymphocytic infiltration, apoptotic cells, and autophagy cells, a semiautomatic cell-counting algorithm for ImageJ was used on eleven spinal cord cross-sectional images per animal, which had distinct distances from the lesion epicenter (0 µm,  ±240 µm,  ±480 µm, ±720 µm, ±960 µm, and  ±1200 µm), a method described in our previous works. The number of positively stained cells was divided by the area of interest, averaged within each animal and displayed as cell density (cells/mm^2^).

### 2.7. Statistical Analysis

For the statistical comparisons of the BBB, gridwalk test, and CatWalk Walk XT^®^ results between groups and time points, two-way repeated-measures ANOVA, followed by Tukey’s test for multiple comparisons, was conducted. The normality assumption was confirmed prior to all the parametric analyses via Shapiro–Wilk normality tests, and a *p*-value of <0.05 was considered significant. Results are presented as the mean ± standard error of the mean (SEM). We acknowledge the limitation in using parametric tests despite the ordinal nature of the BBB scale.

Means between multiple groups in the IHC assessment were analyzed via unpaired *t*-test or one-way ANOVA followed by post hoc Tukey-HSD tests. The normality assumption was confirmed prior to all the parametric analyses via Shapiro–Wilk normality tests, and a *p* value of <0.05 was considered significant. Results are presented as the mean ± standard error of the mean (SEM). All statistical analyses were performed via Prism software (version 9.0, GraphPad Software, Boston, MA, USA). Group sizes were determined a priori by power analysis based on the BBB locomotor score.

## 3. Results

### 3.1. Activation of the Shh Signaling Pathway Following 7-Day Shh Administration

**Figure 3:**
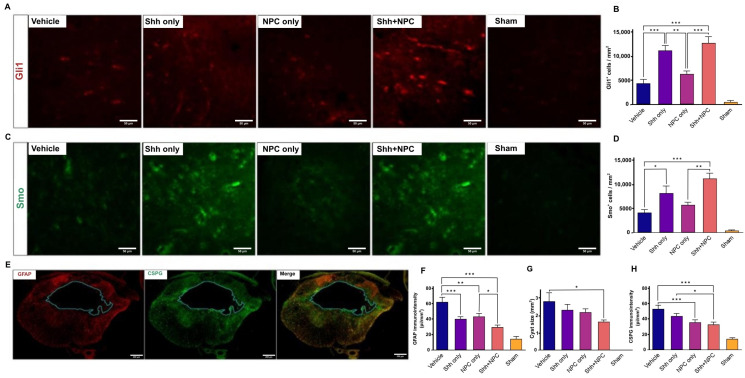
Spinal cord cross-sections from Vehicle, Shh, NPC, Shh + NPC, and Sham animals at 40× magnification stained for GLI1 (**A**) and SMO (**C**) expression. Mean density (cells/mm^2^) of GLI1+ cells (**B**) and SMO+ cells (**D**) in the spinal cord six weeks after injury. Mean immunointensities of GFAP ((**E**), red; (**F**)) and CSPG ((**E**), green; (**H**)) in the spinal cord cross-section of a vehicle-treated animal six weeks after injury. The cyan outline indicates the posttraumatic cyst size (mm^2^) (**G**) after SCI (*n* = 8 for SCI groups (Vehicle, Shh, NPC, Shh + NPC); *n* = 5 for Sham; one-way ANOVA followed by post hoc Tukey-HSD test; *** *p* < 0.001; ** *p* < 0.01; * *p* < 0.05). Scale bar: 50 μm (**A**,**C**), 200 μm (**E**).

After the intrathecal administration of Shh for seven days, we examined the expression of GLI1 and SMO within the spinal cord to verify Shh pathway activation (Figure 3A–D). Six weeks post SCI, GLI1^+^ cell density was highest in the Shh + NPC group (12,879 ± 1255 cells/mm^2^), followed by the Shh-only group (11,303 ± 980 cells/mm^2^), the NPC-only group (6435 ± 565 cells/mm^2^), the vehicle group (4518 ± 669 cells/mm^2^), and the sham group (637 ± 140 cells/mm^2^). There was a significant increase in GLI1 activation in the groups that were administered Shh compared with those that were not (Shh vs. vehicle and Shh + NPC vs. NPC, *p* < 0.001; Figure 3B). Similarly, the density of SMO^+^ cells exhibited analogous results. There was a notable increase in SMO^+^ expression following Shh administration (Shh group vs. vehicle group, *p* = 0.03; Shh + NPC group vs. NPC group, *p* = 0.002; Figure 3D). We hereby verify the downstream activation of the Shh pathway via the exogenous local application of recombinant Shh.

### 3.2. Differentiation of NPCs Following Transplantation into the Injured Spinal Cord

Six weeks post-injury, we aimed to investigate NPC differentiation in vivo and assess whether a 7-day administration of Shh would aid in preserving grafted NPC populations controlled by a placebo.

**Figure 4:**
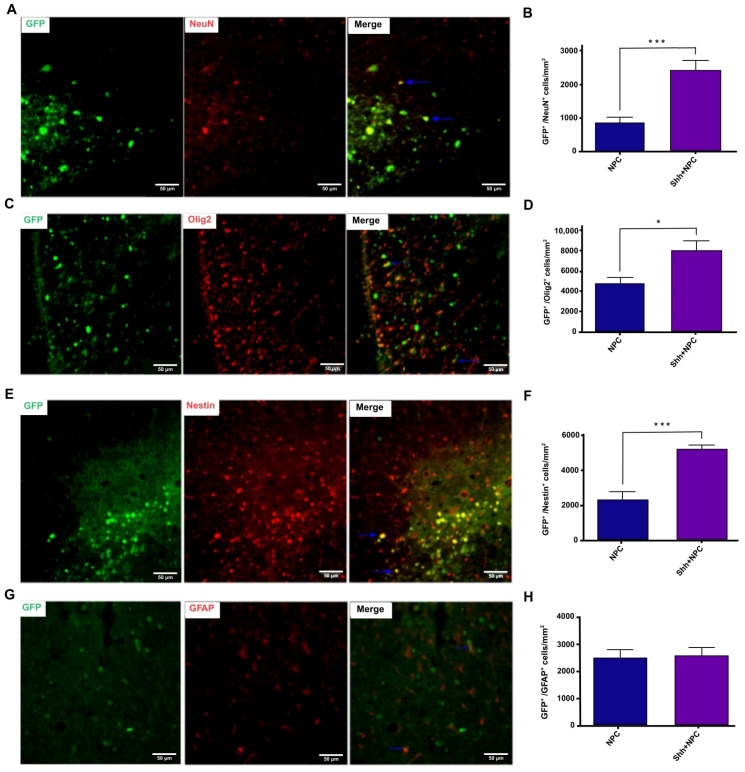
Spinal cord cross-sections of a Shh + NPC-treated animal at 40× magnification stained for NeuN (**A**), Olig2 (**C**), Nestin (**E**), and GFAP (**G**). Mean cell density (cells/mm^2^) of NPC-derived neurons (**B**), oligodendrocytes (**D**), undifferentiated cells (**F**), and astrocytes (**H**) in the spinal cord six weeks after injury. (*n* = 8 for Vehicle, Shh, NPC, Shh + NPC; *n* = 5 for Sham; one-way ANOVA followed by post hoc Tukey-HSD test; *** *p* < 0.001; * *p* < 0.05). Scale bar: 50 μm.

The analysis of GFP-positive and the mature neuron marker NeuN-positive cells (GFP^+^/NeuN^+^) revealed that Shh administration resulted in a significantly greater number of NPC-derived NeuN^+^ mature neurons in the Shh + NPC group (2467 ± 258 GFP^+^/NeuN^+^ cells/mm^2^) than in the NPC group (880 ± 144 GFP^+^/NeuN^+^ cells/mm^2^; *p* < 0.001; Figure 4B).

In the same manner, we evaluated the co-localization of the mature oligodendrocyte marker Olig2 and GFP (GFP^+^/Olig2^+^) to evaluate the differentiation of transplanted NPCs into mature oligodendrocytes. Six weeks post-injury, animals in the Shh + NPC group had significantly more GFP^+^/Olig2^+^ cells/mm^2^ and thus NPC-derived mature oligodendrocytes compared to the NPC group (8101 ± 877 GFP^+^/Olig2^+^ cells/mm^2^ vs. 4800 ± 582 GFP^+^/Olig2^+^ cells/mm^2^; *p* = 0.011; Figure 4D).

To assess the number of transplanted NPCs that remained in an undifferentiated state, we quantified the cell density of GFP^+^/Nestin^+^ cells. There were significantly more GFP^+^/Nestin^+^ NPCs in the Shh + NPC group compared to the NPC group (5248 ± 214 GFP^+^/Nestin^+^ cells/mm^2^ vs. 2380 ± 420 GFP^+^/Nestin^+^ cells/mm^2^; *p* < 0.001; Figure 4F). We interpret these results as an indication of improved survival rate of transplanted NPCs.

In a similar manner, we analyzed NPC differentiation into astrocytes by quantifying GFP^+^/GFAP^+^ cell density. Unlike the previous three populations, the number of NPC-derived astrocytes did not significantly increase by applying Shh (Shh + NPC: 2595 ± 265 cells/mm^2^ vs. NPC-only: 2498 ± 292 cells/mm^2^, *p* = 0.81; Figure 4H).

These findings suggest that the intrathecal administration of Shh promotes the differentiation of transplanted NPCs toward neuronal and oligodendrocytic lineages, rather than the astrocytic lineage, in the injured spinal cord. Concurrently, more NPCs survived and remained undifferentiated at six weeks post transplantation in the Shh-treated animals compared to NPC-only animals.

### 3.3. Effects of NPC Transplantation and Shh Treatment on Glial Scarring, Inflammation, and Cell Death

Reactive astrogliosis is a critical pathophysiological process following SCI that not only inhibits the sprouting and regeneration of endogenous axons but also hinders the integration of transplanted NPCs. In this study, we evaluated reactive astrogliosis in the spinal cord six weeks post SCI by measuring the immunointensity of GFAP across all groups. We observed a significant decrease in GFAP immunointensity in the Shh group (41.05 ± 2.26 pi/mm^2^, *p* < 0.001), the NPC group (44.28 ± 3.04 pi/mm^2^, *p* = 0.002), and the Shh + NPC group (30.43 ± 1.99 pi/mm^2^, *p* < 0.001) compared to the vehicle group (62.37 ± 6.31 pi/mm^2^, Figure 3F). Additionally, astrogliosis in the Shh + NPC group was significantly lower than that in the NPC group (30.43 ± 1.99 pi/mm^2^ vs. 44.28 ± 3.04 pi/mm^2^, *p* = 0.03). As anticipated, uninjured sham animals in the sham group exhibited the least amount of GFAP immunointensity (16.22 ± 1.45 pi/mm^2^) among all (Shh + NPC group *p* = 0.0320; NPC group *p* < 0.001; Shh group *p* < 0.001; vehicle group *p* < 0.001).

The size of the posttraumatic intramedullary cyst was assessed across all eleven GFAP-stained cross-sections (0 µm, ± 240 µm, ± 480 µm, ± 720 µm, ± 960 µm, and ± 1200 µm from the lesion epicenter). Posttraumatic cysts were observed in all injured animals, with the largest area in the vehicle group (2.84 ± 0.47 mm^2^), followed by the NPC (2.22 ± 0.18 mm^2^), Shh (2.34 ± 0.28 mm^2^), and Shh + NPC (1.68 ± 0.06 mm^2^) groups. No visible cysts were detected in the sham group. A significant reduction in cyst size was noted only between the combined treatment group (Shh + NPC) and the vehicle group (*p* = 0.04, Figure 3G).

The deposition of chondroitin sulfate proteoglycans (CSPGs) in the extracellular matrix following SCI contributes to fibrous scar formation, obstructing axonal growth and neural circuit rewiring. Similar to the changes in astrogliosis, we observed that CSPG deposition was significantly lower in the NPC group (36.59 ± 2.56 pi/mm^2^, *p* < 0.001) and the Shh + NPC group (33.64 ± 2.76 pi/mm^2^, *p* < 0.001) compared to the vehicle group (53.99 ± 3.50 pi/mm^2^, Figure 3H).

**Figure 5:**
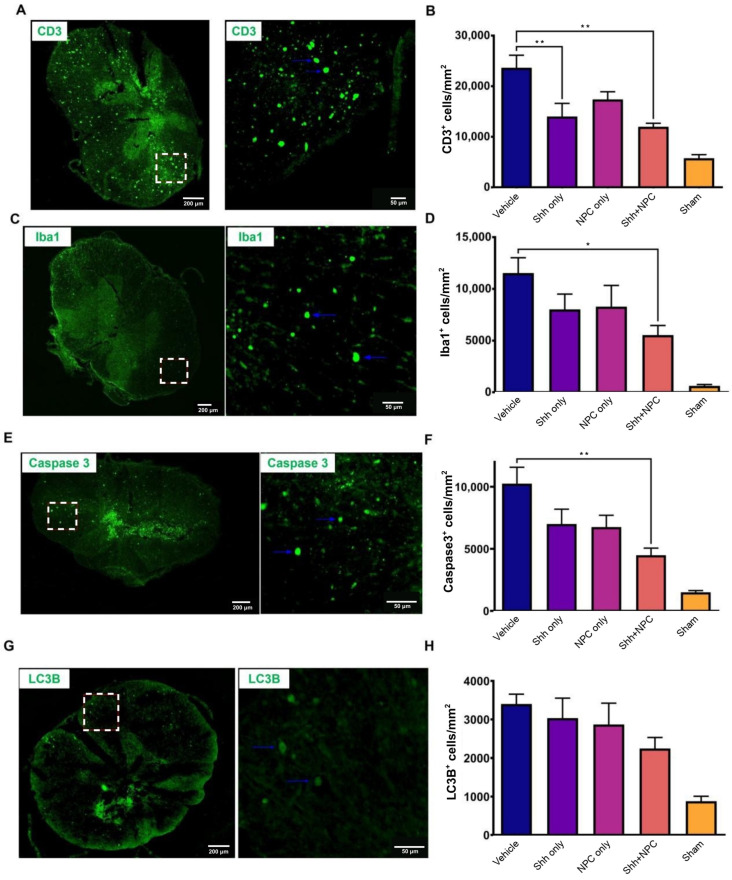
Spinal cord cross-sections at 10× (Scale bar: 200 μm) and 40× (Scale bar: 50 μm) magnification stained for CD3 (**A**), Iba1 (**C**), Cleaved Caspase-3 (**E**), and LC3B (**G**) expression. Mean density (cells/mm^2^) of CD3^+^ cells (**B**), Iba1+ cells (**D**), Cleaved Caspase-3^+^ cells (**F**), and LC3B+ cells (**H**) in the spinal cord six weeks after injury. (*n* = 8 for SCI groups (Vehicle, Shh, NPC, Shh + NPC); *n* = 5 for Sham; one-way ANOVA followed by post hoc Tukey-HSD test; ** *p* < 0.01; * *p* < 0.05).

Regarding T-lymphocyte infiltration as a marker of post-injury immune activation, we quantified the number of CD3^+^ cells, which are present only in a minor amount in the spinal cord under physiological conditions. The highest density of T-lymphocytes was observed in the vehicle group (23,648 ± 2480 CD3+ cells/mm^2^), followed by the Shh + NPC group (17,387 ± 1515 CD3+ cells/mm^2^), the Shh group (14,000 ± 2597 CD3+ cells/mm^2^), and the NPC group (11,641 ± 2510 CD3+ cells/mm^2^). Compared to vehicle treatment, combined treatment with Shh + NPC, as well as Shh-only treatment, resulted in a significant reduction in T-lymphocyte infiltration (*p* = 0.001 and *p* = 0.008, Figure 5B).

Staining for Iba1, a general marker for various sublineages of macrophages, indicates either macrophage infiltration or resident microglial activation in the CNS. Notably, only the combined treatment in the Shh + NPC group (5531 ± 930 cells/mm^2^) resulted in a significantly lower macrophage density than did the vehicle (11,532 ± 1479 cells/mm^2^, *p* = 0.04; Figure 5D). Effects in the Shh- and NPC-only groups remained insignificant.

The presence of the apoptotic marker cleaved Caspase-3, which is indicative of controlled cell death as part of the secondary injury cascade after SCI, was assessed by quantifying the cleaved Caspase-3^+^ cell density at six weeks post SCI. A significant difference in apoptotic cell density was observed between the vehicle (10,251 ± 1321 cells/mm^2^) and the Shh + NPC groups (4486 ± 572 cells/mm^2^, *p* = 0.002; Figure 5F).

The microtubule-associated protein 1A/1B light chain 3B (LC3B), a central protein in the autophagy pathway, serves as a surrogate indicator of autophagy activity. The evaluation of LC3B+ cell density in spinal cord cross-sections at six weeks post-injury revealed that all four SCI groups presented greater autophagy activity compared to the sham group, but the differences among the different interventions remained statistically insignificant (Figure 5H).

These findings suggest that activation of the Shh pathway and the local effects of grafted NPCs separately reduce immune-cell migration and persistent inflammation compared with controls. Moreover, these effects are significantly stronger in the combined Shh + NPC treatment.

### 3.4. Synergistic Effects of NPC and Shh Treatment on Functional Motor Recovery

To assess locomotor recovery after injury and treatment, we subjected all rodents to functional tests on a weekly basis. We used observation-based scores (BBB, gridwalk), as well as computerized gait analysis (CatWalk XT^®^) to measure different aspects of locomotor recovery.

**Figure 6:**
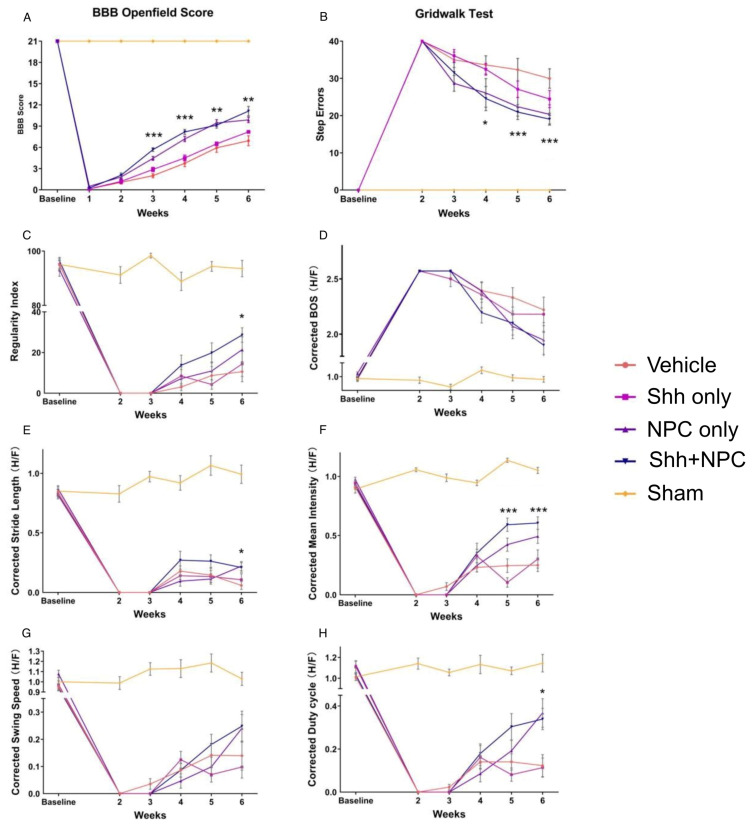
(**A**) BBB open field scores of the animals from baseline until six weeks after SCI/sham surgery displayed in mean ± SEM. (**B**) Number of stepping errors in the gridwalk test at baseline until six weeks after SCI/sham surgery, displayed as mean ± SEM. (**C**) Regularity index, (**D**) base of support, (**E**) stride length, (**F**) mean intensity, (**G**) swing speed, and (**H**) duty cycle of the hindlimbs as CatWalk XT^®^ parameters at baseline until six weeks after SCI/sham surgery displayed as mean ± SEM (*n* = 8 for Vehicle, Shh, NPC, Shh + NPC; *n* = 5 for Sham; two-way repeated measures ANOVAs, followed by Tukey tests for multiple comparisons; * *p* < 0.05, ** *p* < 0.01, *** *p* < 0.001; asterisks denote significant pairwise differences between the Shh + NPC group and the Vehicle group in all panels).

In the BBB open field test, the recovery of hindlimb movement was evaluated via a 21-point score at baseline and weekly after SCI until the end of the experiment. Three weeks post-injury, the motor function of the Shh + NPC group (5.69 ± 0.27) improved compared with the vehicle group (2.0 ± 0.28, *p* < 0.001), the Shh group (2.88 ± 0.31, *p* = 0.02), and the NPC group (4.44 ± 0.27, *p* = 0.04). Compared with vehicle, NPC-only-treated animals also demonstrated significant improvement (*p* < 0.001). This trend persisted until the end of the experiment at week 6, with the NPC and Shh + NPC groups continuing to show superior locomotor recovery compared with the Shh-only and vehicle groups (Figure 6A). At the end of the experiment, the Shh + NPC group achieved an average score of >11 points, indicating full weight-bearing capacity and the onset of occasional interlimb coordination, whereas the vehicle and Shh groups could not reach 9 points, indicating an inability to support their body weight (Figure 6A).

The gridwalk test is an advanced behavioral test that emphasizes coordination, fine motor skills and hind limb strength. Two weeks post-injury, all injured animals were unable to bear weight and were therefore counted as stepping errors throughout, preventing intergroup comparisons. Starting from week four post-injury, the Shh + NPC group (24.62 ± 3.10 step errors) presented a significant reduction in the number of stepping errors compared with the Shh group (32.46 ± 1.59 step errors, *p* = 0.03) and the vehicle group (33.63 ± 2.45 step errors, *p* = 0.02). Six weeks post-injury, the difference in stepping errors between the Shh + NPC group (19.04 ± 1.62) and the NPC group (20.33 ± 2.56, *p* > 0.99), as well as the Shh group (24.46 ± 2.31, *p* = 0.36), was diminished. All treatment groups showed significantly fewer stepping errors compared to the vehicle group (29.96 ± 2.61), especially compared with the Shh + NPC group (*p* < 0.001; Figure 6B).

The CatWalk XT^®^ gait analysis is a comprehensive and objective method that involves evaluating multiple aspects of locomotor function, including muscle tone, paw measurement, weight support, coordination, and walking patterns. We conducted the CatWalk gait analysis at baseline and weekly after SCI until the conclusion of the experiment. Since our T9-SCI model does not affect the front limbs, we neutralized individual animal differences by applying an internal correction, dividing hindlimb parameter values by front limb values, and pooling these corrected values for statistical analysis. The regularity index (RI) measures hindlimb coordination and is defined as the exclusive use of normal step sequence patterns (NSSPs) during uninterrupted locomotion. Gradual recovery of coordination was observed in all SCI animals. However, significant restoration of coordination was found only between the Shh + NPC group (28.63 ± 3.54%) and the vehicle group (10.60 ± 5.00%, *p* = 0.04; Figure 6C) at the end of the experiment. A similar improvement was noted in stride length, which measures the distance between successive placements of the same paw. Six weeks post-injury, the corrected stride length in the Shh + NPC group recovered from 0 (second week post-SCI) to 0.21 ± 0.04, a significant improvement compared with the vehicle group (0.06 ± 0.03, *p* = 0.047; Figure 6E). Additionally, the duty cycle, which expresses the stand phase as a percentage of the time between two consecutive initial contacts of the same paw, improved significantly in the Shh + NPC group at six weeks post SCI, increasing to 0.34 ± 0.05, which was significantly greater than that in the vehicle group (0.12 ± 0.05, *p* = 0.03) and the Shh group (0.11 ± 0.05, *p* = 0.01; Figure 6H). Notable improvements were not observed in other key kinematic CatWalk parameters, such as the base of support (the average width between the hind paws, Figure 6D) and swing speed (the speed of the paw during a swing, Figure 6G). However, as a more static parameter indicating weight-bearing ability and muscle tone, the corrected mean intensity was associated with earlier motor recovery in the Shh + NPC group than in the vehicle group (five- and six-week post-SCI: *p* < 0.001, Figure 6F).

## 4. Discussion

NPC transplantation remains a promising therapeutic approach for treating SCI, as supported by prior studies demonstrating the capacity of NPCs to differentiate into neurons and oligodendrocytes, integrate into host tissue, promote axonal regeneration, and contribute to functional recovery [9]. Despite these advantages, the efficacy of NPC-based therapies is limited by poor post-transplantation survival and restricted differentiation, largely due to the hostile posttraumatic microenvironment. Our present study aimed to increase the survival and integration of NPCs through the adjunctive application of Shh, a signaling protein that is critically involved in neural development, which is also known for its proliferative and patterning effects on neural precursors.

Previous in vitro work from our group demonstrated that Shh application promotes NPC proliferation and neuronal differentiation under inflammatory stress, mimicking subacute post-injury conditions [24]. In this in vivo investigation, we extended those findings by combining NPC transplantation with continuous Shh administration via intrathecal osmotic micropumps for seven days, a delivery protocol adapted from the methodology of Bambakidis and colleagues [21,28]. Unlike single-dose injections or vector-based delivery systems, this approach allows for sustained local exposure while avoiding systemic side effects. This strategy also circumvents the risks associated with the potent mitogenic effects of Shh, which include the potential for uncontrolled proliferation and tumorigenesis, as observed in contexts such as polydactyly, solid tumors, and hematological malignancies [29,30]. For these reasons, we deliberately avoided systemic or viral delivery, opting instead for spatially and temporally controlled local application.

While the exact intrathecal Shh concentrations in the lesioned spinal cord were not directly measured, immunohistochemical analysis revealed significant upregulation of the Shh pathway in treated animals. Notably, the expression levels of SMO and GLI1, two canonical markers of pathway activation, were markedly elevated in both the Shh-only and Shh + NPC treatment groups. These findings confirmed that local Shh delivery successfully activated downstream signaling pathways and validated the effectiveness of the applied delivery protocol. The intrathecal Shh dose was adopted from Bambakidis et al. [19,26] and established in our previous experiments. The transplanted graft size (4 × 10^5^ GFP + NPCs) also followed our previously established protocol [8,9].

Functionally, the addition of Shh to NPCs significantly increased the proportion of surviving NPCs five weeks after grafting. We were able to observe nearly twice the density of undifferentiated GFP^+^/Nestin^+^ cells in the Shh + NPC group compared with the NPC-only group. Furthermore, Shh enhanced the differentiation of transplanted cells into mature oligodendrocytes and neurons, as indicated by elevated numbers of GFP^+^/Olig2^+^ and GFP^+^/NeuN^+^ cells. These results suggest that Shh not only supports cell survival but also promotes lineage-specific maturation, which aligns with previous studies highlighting the role of Shh in neural lineage specification [31]. In contrast, the density of NPC-derived astrocytes (GFP^+^/GFAP^+^) remained unchanged between the NPC-only and Shh + NPC groups, indicating a selective effect of Shh on neuronal and oligodendrocytic, but not astrocytic, differentiation.

Interestingly, while NPC-derived astrocyte numbers were similar across groups, Shh-treated animals exhibited a marked reduction in overall reactive astrogliosis, as reflected by significantly reduced GFAP immunoreactivity. These findings suggest that Shh modulates host astrocyte responses and may attenuate the formation of inhibitory glial scars, a well-documented barrier to neural regeneration. Notably, the source of Shh in the injured CNS remains under debate, with neurons proposed as primary producers and astrocytes potentially acting as either source or recipient cells [18]. Recent nomenclature efforts emphasize the complexity of astrocyte responses, underscoring the context-dependent spectrum of activation states [32]. Thus, the observed reduction in astrogliosis may reflect a shift toward a neuroprotective astrocyte phenotype, possibly facilitated by Shh signaling.

It should be noted, however, that GFAP is not exclusively expressed by mature reactive astrocytes. Immature neural precursor cells also express GFAP, potentially confounding GFAP immunointensity measurements in animals that received NPC transplants. Future studies should consider ALDH1L1, a pan-astrocyte marker shown to label a substantially broader and more specific astrocytic population than GFAP [33,34].

Several studies have highlighted the multifaceted role of Shh in SCI and other CNS injuries, reporting its involvement in neurogenesis, remyelination, axonal guidance, and immune response modulation [23,35,36,37]. These diverse functions further support its application as an adjunct to cell-based therapies, although dosage and delivery must be carefully controlled to avoid adverse effects.

The combined treatment also led to a significant decrease in the post-traumatic intramedullary cyst, an important hallmark of chronic SCI that impedes tissue continuity and axonal regeneration. In addition, decreased deposition of chondroitin sulfate proteoglycans (CSPGs) in the extracellular matrix was observed, further supporting an environment conducive to axonal outgrowth. It should be noted, however, that the antibody used to detect CSPGs (MAB1581, Millipore) targets the core protein of aggrecan specifically and does not recognize other CSPG molecules such as phosphacan or neurocan. As such, the immunohistochemical signal reflects changes in aggrecan expression rather than total CSPG content, which represents a limitation of the current study.

Importantly, Shh + NPC-treated animals presented fewer CD3+ T-lymphocytes and reduced Iba1+ macrophage infiltration and microglial activation, suggesting mitigation of the post-injury inflammatory response. While NPCs or Shh alone did not significantly affect T-cell density, the combination of both was necessary to elicit this effect. Given the role of T-cell infiltration in trauma-induced autoimmunity [38], this observation may hold relevance for long-term outcomes.

Apoptosis, as indicated by cleaved Caspase-3 expression, was significantly reduced in the combined treatment group, suggesting that Shh and NPCs synergistically alleviate secondary injury cascades. Interestingly, while all the SCI groups presented greater autophagy activity than the sham group did, no significant intergroup differences in LC3B expression were detected, suggesting that the beneficial effects of Shh and NPCs may not be mediated through autophagic modulation [39,40]. The improved histological outcomes are likely due to attenuation of the neuroinflammatory and apoptotic milieu that otherwise limits regeneration.

These histological findings are accompanied by measurable functional benefits. The BBB locomotor scores clearly improved in the NPC-only and combined treatment groups from week three onward. While Shh alone modestly reduced astrogliosis and immune cell infiltration, it failed to elicit functional gains on its own. In contrast, Shh + NPC-treated animals showed superior functional performance in the BBB test and gridwalk task, with fewer stepping errors and improved coordination by week six. Although CatWalk XT^®^ gait analysis revealed only minor differences in dynamic parameters such as stride length and swing speed, static indicators—particularly corrected mean intensity—reflected earlier and sustained recovery in the Shh + NPC group. This observation aligns with previous studies showing that CatWalk XT^®^ parameters are less sensitive in animals that have not regained full weight-bearing ability [41,42] and underscores the continued relevance of combining multiple behavioral metrics in SCI studies [43].

Several limitations of this study should be acknowledged. Firstly, only female rats were used, following the prevailing convention in preclinical SCI research. Among other reasons, the female urethral anatomy permits reliable, atraumatic manual bladder expression and lowers post-operative morbidity and mortality after SCI [44]. Biological sex can modulate recovery, as is confirmed in SCI studies comparing male and female rodents [45,46].

Furthermore, we used minocycline and cyclosporine—with known immunomodulatory and neuroprotective properties—in our post-operative care protocols. They were administered identically to all injured groups and therefore form a uniform pharmacological background that cannot account for the between-group differences reported here.

Lastly, our functional assessment was restricted to motor outcomes (BBB, Gridwalk, CatWalk). Although no overt neuropathic pain or autotomy was observed, dedicated sensory testing (e.g., von Frey, Hargreaves) was not performed and remains an important objective for future work.

As outlined before, the injured cord passes through distinct temporal phases [11,12,13], and we timed grafting to exploit this biology rather than for surgical convenience. We selected the subacute window (~day 7), when the acute cytotoxic and inflammatory response is subsiding and shifting towards pro-repair macrophage activity and active endogenous trophic signaling. Although there is injury, the tissue remains plastic, and the dense astroglial–fibrotic scar has not yet consolidated. Grafting into this permissive interval avoids both the hostile acute milieu and the mature, glial barrier of the chronic SCI, each of which compromises NPC graft survival and integration. This choice is consistent with accumulating evidence that the subacute phase is the optimal transplantation window for NPCs, conferring superior survival, migration and locomotor recovery relative to acute or chronic grafting [47,48,49].

The present study assessed outcomes over a six-week period, which reflects the subacute phase of SCI. While this timeframe is sufficient to capture the initial regenerative and anti-inflammatory effects of the combined treatment, the long-term implications of Shh + NPC therapy remain to be established. On the beneficial side, sustained NPC-derived neuronal and oligodendroglial integration could theoretically contribute to progressive circuit remodeling and continued functional improvement. On the risk side, the mitogenic properties of Shh signaling raise the concern that prolonged pathway activation could promote aberrant cell proliferation. Although no obvious histological evidence of tumor formation or excessive proliferation was observed at six weeks, we cannot exclude changes. Long-term safety studies extending beyond the subacute phase will be necessary before clinical translation can be considered.

## 5. Conclusions

In summary, our study demonstrated that intrathecal Shh delivery enhances the efficacy of NPC transplantation in a rodent model of thoracic SCI. The combined therapy promoted NPC survival, favored differentiation into neuronal and oligodendroglial lineages, and ameliorated several secondary injury features, including glial scarring, cyst formation, immune cell infiltration, and apoptosis. These changes correlated with significant improvements in motor function. Collectively, our findings highlight the potential of Shh as a powerful adjunct to cell-based therapies and advocate for continued exploration of synergistic combinatorial approaches in the treatment of SCI.

## Figures and Tables

**Table 1:** Group design.

	Group	Animal Number (*n*)	T9 SCI	Shh Application	NPC Transplantation
Group 1	Vehicle	8	+	−	−
Group 2	Shh-only	8	+	+	−
Group 3	NPC-only	8	+	−	+
Group 4	Shh + NPC	8	+	+	+
Group 5	Sham	5	−	−	−

## Data Availability

All relevant data are contained within the article. The original contributions presented in the study are included in the article, and further inquiries can be directed to the corresponding author.
